# Comparative Proteome Identifies Complement Component 3-Mediated Immune Response as Key Difference of Colon Adenocarcinoma and Rectal Adenocarcinoma

**DOI:** 10.3389/fonc.2020.617890

**Published:** 2021-02-15

**Authors:** Jun-Ze Liang, Xiao-Ling Liang, Li-Ye Zhong, Chu-Tian Wu, Jing Zhang, Yang Wang

**Affiliations:** ^1^MOE Key Laboratory of Tumor Molecular Biology and Key Laboratory of Functional Protein Research of Guangdong Higher Education Institutes, College of Life Science and Technology, Institute of Life and Health Engineering, Jinan University, Guangzhou, China; ^2^Department of Gastroenterology, The First Affiliated Hospital, Jinan University, Guangzhou, China

**Keywords:** rectal adenocarcinoma, proteome, immune response, complement component 3, colon adenocarcinoma

## Abstract

Colorectal cancer (CRC) is one of the most lethal diseases with high morbidity and mortality worldwide. Clinically, tumors located in colon and rectum have diverse prognosis and therapeutic outcome. Here, we performed data mining derived from 20 CRC patient samples to compare proteomic difference between colon adenocarcinoma (COAD) and rectal adenocarcinoma (READ). We found that differential expressed proteins (DEPs) upregulated in COAD were mainly enriched in immune response, moreover, higher immune scores were found in COAD than READ, as calculated by The Cancer Genome Atlas (TCGA) data. To identify the core protein of DEPs with high prognostic value for COAD, we performed topological overlap matrix (TOM) to investigate the hub proteins using 77 immune-relevant DEPs, and identified complement component 3 (C3) as the core protein in the immune-relevant DEPs matrix between the COAD and READ. Moreover, we found that C3 was up-regulated in COAD, and its expression was negatively associated with overall survival of COAD patients but not READ. In conclusion, we identified C3-mediated immune response as key feature to distinguish COAD and READ, and highlighted C3 as potential biomarker with high prognostic value for clinical application, which provided new clue for precise treatment of COAD.

## Introduction

Colorectal cancer (CRC) is the third most widespread carcinoma and the fourth deadliest cancer at present, with continuously increasing number of patients in the past few years. Although CRC death rates are slowly decrease in worldwide (1), the five‐year overall survival for patients remains poor. Approximately half of CRC patients die from their cancer (2). The survival rate of CRC remains far from satisfactory due to its late diagnosis, rapid development and easy metastasis despite considerable advancements in therapeutic strategies have been achieved (3). Therefore, extensive and in-depth studies are needed for improvement in diagnosis, treatment of CRC and the prediction of its recurrence. Since colon adenocarcinoma (COAD) and rectal adenocarcinoma (READ) were historically treated as homogeneity, they have long been considered as a single cancer type for therapy in clinical. Accumulating studies showed that COAD and READ have diverse metastatic patterns, spread ratio and drug response in patients (4), suggesting COAD and READ can be deemed as two distinct cancers for clinical treatment. Thus, it is necessary to identify more molecular characteristics to distinguish them, which provides clues for their clinical treatment respectively.

Previously, the genetic and epigenetic distinctions of COAD and READ had been investigated. Some mutations on BRAF, CTNNB1, PIK3R1 were more frequently observed in COAD while mutations on APC, ERBB2, STK11, and TP53 were always found in READ. These genetical differences could only explain some malignant behaviors of COAD and READ, however, their differences on protein level remained unclear. As the “executioners of life”, proteins determine cell phenotype, which means single level of nuclear acid can’t explain the biological variation comprehensively. Recent study suggested that nuclear acid-based genomic data did not fully consist with proteomic data (5). Current proteomic analysis afforded a new paradigm for understanding cancer biology with functional context to interpret genomic data (6). Thus, it is critically important to profile specific biomarkers to distinguish COAD and READ for better understanding of these two diseases and supporting the tailoring of medical treatment.

In this study, we profiled the protein difference between 20 cases of COAD and READ, and identified the immune response including complement and coagulation system, as the key distinction that specifically up-regulated in COAD. Immune score was performed to evaluate immune activity of COAD and READ. Among DEPs, C3, a crucial protein with the highest centrality, was identified as a potential therapeutic target for COAD therapy.

## Method

### Data Retrieving

We downloaded protein expression RAW files (15 fractions/sample) and clinical information of 40 CRC patients with pathologic diagnosis from ProteomExchange database (PXD015905) (7). These samples included 20 COAD and 20 READ. In addition, 38 transcriptome FPKM files containing 18 samples of COAD and 20 samples of READ were downloaded from TCGA database. The extend mRNA expression files containing 127 COAD and 66 READ samples were downloaded by R package “RTCGA.mRNA”. The Immunohistochemistry (IHC) staining of C3 in COAD and READ tissue slices stained by CAB004209 antibody was compared by using Human Protein Atlas, the IHC intensity was quantified by Image J software and plug-in IHC Profiler.

### Database Searching

The protein expression RAW files were searched against the reviewed protein FASTA file of homo sapiens from Uniprot by using Maxquant (1.6.15.0). Instrument model was Obitrap type and the function of match between runs was set to recalibration. Enzyme mode was trypsin while digestion principle was specific digestion. Oxidation(M) and acetylation (N-Terminal) were selected as variable modification while carbamidomethyl (C) was fixed modification. In the perspective of identifying the protein groups, each protein group should have at least one unique peptide, two unique + razor peptides, and two peptides (8). We chose revert decoy database to calculate FDR. The three parameters named “PSM FDR”, “Protein FDR”, and “Site decoy fraction” were set to 0.01, and the quantification strategy was label free. For protein quantification, the protein abundance was compared by using Label Free Quantification (LFQ) intensities calculated by Maxquant software.

### Clustering and Statistical Analysis

The characteristic characters of 40 CRC patients were clustered into different clusters through binary variable distance and hierarchical clustering by R studio. Three characters containing gender, tumor type and tumor site were the variables of the analysis. The graph displaying the analysis was generated by the R packages of “dendextend”, “ape”, “ggplot2”, “RColorBrewer”, “ggtree”.

PCA is an algorithm to reduce dimensionality of data while retaining most of the variation. By using the proteomic data, we distinguished COAD and READ by two principal components with the highest percentage of explained variation. Then, we calculated the contribution value of DEPs to principal components to find hub protein in the dataset.

### Kyoto Encyclopedia of Genes and Genomes (KEGG) and Gene Ontology (GO) Analysis

KEGG and GO analysis were performed using R packages of “clusterProfiler” (9) and “org.Hs.eg.db”. Moreover, significantly DEPs enriched to different subcellular locations were generated by R packages. Fisher’s exact test was used to calculate the *P*-value of enrichment analysis.

### Immune Scores and Immune Cells’ Markers Enrichment Using Single Sample Gene Set Enrichment Analysis (ssGSEA)

Immune gene sets respectively supplied by Yoshihara K and Charoentong P were used to calculate the immune scores and the enrichment scores of immune cells’ markers. The core of immune scores and immune cells’ markers enrichment were ssGSEA and they were calculated by “estimate” and “GSVA R packages”. Immune scores referred to the immune cell admixture and the enrichment scores of immune cells’ markers, which indicated the infiltration of immune cells.

### Network of Topological Overlap Matrix (TOM)

The R packages “WGCNA” (10), “ggraph”, “igraph”, and “tidygraph” were used to establish TOM of 77 DEPs. Power of 5 were set to build TOM, as it was the closest approximation to non-scale network, and had high mean connectivity in TOM. According to the dissimilarity, 77 DEPs were divided into two modules, respectively, the 62 DEPs in blue module were used to drawn the interacted network.

### Sectionalized Imputation

Mean_all_ was the mean and Sd_all_ was the standard deviation of the whole expression matrix. The protein variables containing missing values over 16 in both COAD and READ group were discarded. Then, the protein variables with more than 18 missing values in one group and less than 8 missing values in another was extracted as one group. In the remaining data, only few missing values belonged to missing completely at random (MCAR). The occurrence of mass spectrometry’s missing values was caused by low abundant proteins, and the peak of histogram was less than 0 after normalization. This type of missing values was not suitable for single KNN proximity algorithm, as referred to Cosmic Lazor’s article (10). Hence, it was necessary to classify missing values by threshold of proteins’ abundance and treated them differently. The threshold was defined as follow,

$$Threshold1\;=\;2\;*\;Sd_{all}\;+\;Mean_{all}$$

We used the KNN algorithm (R package of “DMwR”) to impute the remaining missing values. On the one hand, the missing values of proteins’ abundance greater than Threshold1 were used KNN proximity algorithm. On the other hand, the method that missing values of the proteins’ abundance less than Threshold1 was introduced as follow. Because missing values were associated with low abundant proteins, the basic of the method is using array with lower mean and standard deviation than per sample to impute missing values. We defined adjusted standard deviation and mean of per sample. They read *Adj.Sd_sam_* and *Adj.Mean_sam_*,

$$Adj.Sd_{sam}\;=\;0.3\;*\;Sd_{sam}$$

$$Adj.Mean_{sam}\;=\;Mean_{sam}-\left(1.8*Sd_{sam}\right)$$

*Mean_sam_* was the average and *Sd_sam_* is the standard deviation of each sample. Eventually, with the mean of *Adj.Mean_sam_* and the standard deviation of *Adj.Sd_sam_*, the random numbers replacing the missing values in every sample were generated. Finally, detected by root mean square error (RMSE), the method we used was better than single KNN proximity algorithm and filling up 0. The histogram exhibiting the distribution of missing values was drawn by R package named “ggplot2”.

### The Relevant R Packages

R version was 4.0.2 and R studio was used to write program. Totally, we used R packages of “tidyr”, “ggtree” (11), “ggplot2”, “WGCNA”, “clusterProfiler”, “ggraph”, “igraph” “tidygraph”, “circlize”, “ComplexHeatmap”, “Pheatmap”, “estimate”, “DMwR”, “psych”, “reshape2”, “factoextra”, “genefilter”, “GSVA”, “Biobase”, “stringr”, “ggthemes”, “dendextend”, “ape”, “RColorBrewer”, “GOplot”, “Hmisc”, “corrplot”, “RTCGA”, “dplyr” for analysis.

### Protein-Protein Interaction Network and Survival Analysis

The network of protein-protein interaction was performed by REACTOME. The clinical information of patients was generated from CPTAC and TCGA. The mRNA expression datasets were downloaded from TCGA by R package “RTCGA.mRNA”. Survival outcome analysis modeled results in reference to the patient overall survival. The survival curve was downloaded from GEPIA (http://gepia.cancer-pku.cn/index.html).

### Statistical Method

Except screening out the DEPs through proteome, under the condition of homogeneity variance (Levene Test P > 0.1) and satisfying gaussian distribution (Shapiro-Wilk P > 0.1), we used Student Test to test whether the two populations’ means were equal. The Mean-Whitney U test was applied to screen out the DEPs or the case of heterogeneity variance (Levene Test P ≤ 0.1) or unsatisfying gaussian distribution (Shapiro-Wilk P ≤ 0.1).

## Results

### Difference of COAD and READ in Proteome

**Figure 1:**
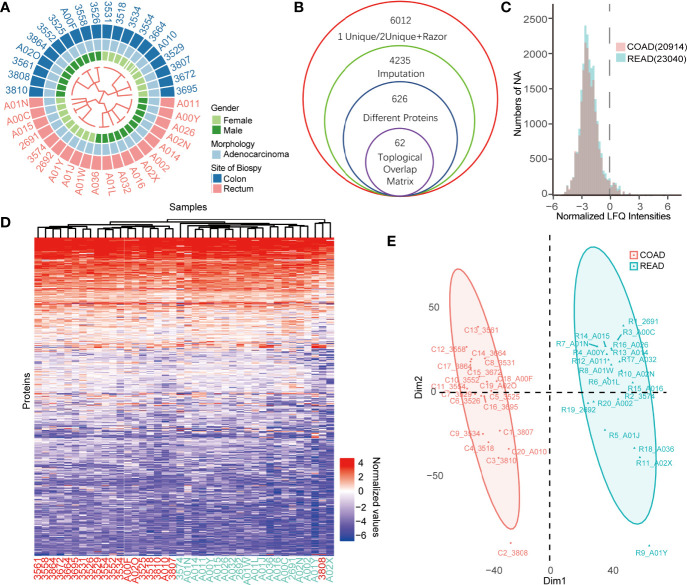
Comparative proteomics analysis of colon adenocarcinoma (COAD) and rectal adenocarcinoma (READ). **(A)** The heatmap exhibited overview of patients’ characters including gender, morphology, site of biopsy using binary cluster. **(B)** The workflow of differential expressed proteins (DEPs) screening was displayed. **(C)** The histogram showed the numbers of missing values of the COAD and READ. **(D, E)** K-means cluster analysis **(D)** and principal component analysis (PCA) **(E)** of COAD and READ showed significant difference of proteins expression profiles between the two cancers.

To identify the prognostic biomarkers between colon cancer and rectal cancer, we collected 40 protein expression datasets derived from 20 COAD and 20 READ tissues for proteomic analysis. Among the 40 patients showed in the **Figure 1A**, 21 cases were males and 19 cases were females. The workflow for conditionally screening out proteins group was showed in **Figure 1B**. By clustering the samples based on LFQ intensities of proteins, a total of 6,012 proteins with FDR less than 0.01 were identified using Maxquant (1.6.15.0). To guarantee the comparability of each values in COAD and READ datasets, we performed sectionalized imputation strategy to impute the missing valves, and found that the distribution of missing values between COAD (20,914) and READ (23,040) were similar (**Figure 1C**). After centralizing LFQ intensities and imputation of missing values, the remained 4,235 proteins, were subjected to differential expressed proteins (DEPs) analysis. Next, our cluster analysis performed by K-Means cluster algorithm showed that protein expression pattern between COAD and READ was significantly different (**Figure 1D**), which was also supported by two dimensions of principal component analysis (PCA) (**Figure 1E**). These results suggested that COAD and READ may be two diverse cancer types with different proteomic pattern.

### COAD and READ Are Different in Immune Activity

**Figure 2:**
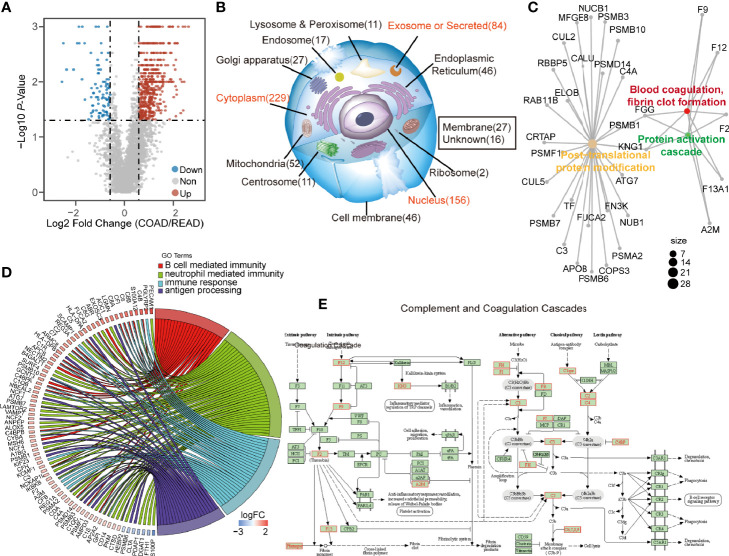
The differential expressed proteins (DEPs) between colon adenocarcinoma (COAD) and rectal adenocarcinoma (READ) enriched in immune activation. **(A)** The volcano graph described 534 up-regulated proteins and 92 down-regulated proteins in COAD. **(B, C)** The DEPs were subjected to subcellular location enrichment analysis and biological pathway enrichment analysis, showing the DEPs were mainly located to secreted proteins, and relevant to the biological progress of PTM, protein activation cascade and blood coagulation, fibrin clot formation. **(D)** The biological pathway enrichment analysis also showed that four immune-relevant pathways including antigen processing and presentation of exogenous peptide antigen, humoral immune response, neutrophil mediated immunity, B cell mediated immunity were upregulated in COAD. **(E)** Kyoto Encyclopedia of Genes and Genomes (KEGG) analysis showing the pathway of complement and coagulation cascade. The identified upregulated proteins in COAD compared to READ were labeled as red, while the downregulated proteins were showed in blue.

Next, we sought to find out the key biological pathways between COAD and READ. The DEPs with the ratio higher than 1.5 or less than 0.67, and p-value less than 0.05, were plotted in volcano plot (**Figure 2A**). A total of 626 DEPs including 534 up-regulated and 92 down-regulated proteins were identified (**Table S1**), which was found to be abundant in cytoplasm, nucleus and exosome, as revealed by cellular component analysis. Interestingly, the DEPs abnormally enriched in extracellular environment (exosome and secreted proteins) raised our attention (**Figure 2B**, **Table S2**). Biological analysis showed that proteins in regulating PTM progression, protein activation cascade, blood coagulation were differently expressed between the two cancers (**Figure 2C**). In addition, biological pathway analysis showed that four immunity-related pathways, including B cell mediated immunity, neutrophil cell immunity, humoral immune response, and antigen processing and presentation of exogenous peptide antigen, were enriched (**Figure 2D**, **Table S3**). Among which, the KEGG enrichment analysis demonstrated that the 534 up-regulated DEPs in COAD were enriched in complement and coagulation cascades (**Figure 2E**). Since immunity was highly associated with extracellular environment, secreted proteins and proteins activating cascades, we speculated that COAD and READ are different in complement system and these relevant proteins may be used as biomarkers for clinical application. Interestingly, the 92 down-regulated DEPs mainly enriched on “Oxidative phosphorylation” and “ATP synthesis coupled electron transprot” (**Figure S1A**). Particularly in COAD, we observed proteins relevant to fatty acid oxidation and glucose catabolic process were upregulated, but oxidative phosphorylation associated proteins were suppression (**Figure S1B**). We speculated that high level of fatty acid oxidation and glucose catabolic process in COAD can suppress oxidative phosphorylation to promote lactic acid production (12), which modulates the tumor microenvironment to promote immune response (13).

### COAD Has Higher Immune Scores Than READ

**Figure 3:**
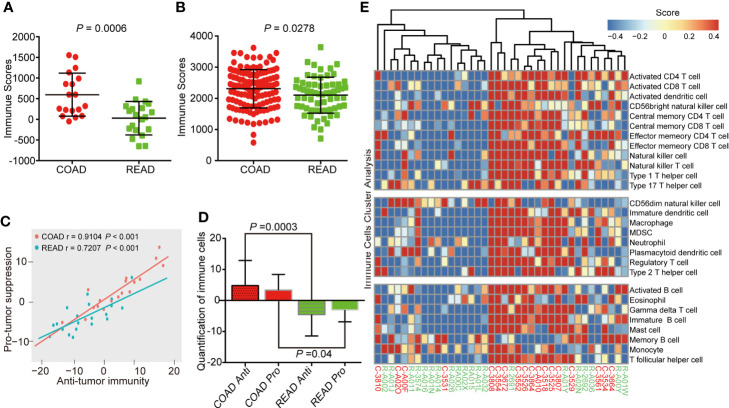
Colon adenocarcinoma (COAD) has higher immune score than rectal adenocarcinoma (READ). **(A)** Immune scores analysis showing that COAD had higher immune scores than READ in protein level, as analyzed by proteomics of 38 clinical samples. **(B)** COAD exhibited higher immune scores than READ in mRNA level of 238 samples (172 COAD and 66 READ). **(C)** Correlation between infiltration of cell types executing anti-tumor immunity and cell types executing pro-tumor, immune suppressive functions in COAD and READ. r coefficient of Pearson’s correlation. **(D)** The quantification of both pro-tumor suppression (Pro) and anti-tumor immunity (Anti) relevant immune cells were both increased in COAD. **(E)** Single-sample gene set enrichment analysis clustering the relative infiltration of immune cell populations for COAD and READ. The relative infiltration of each cell type is normalized into a z-scores.

Immune microenvironment plays an important role in the development of colorectal cancer (14). As a major type of non-tumor components, immune cells were valuable for diagnostic and prognostic assessment of tumors (15). ESTIMATE (Estimation of Stromal and Immune cells in Malignant Tumor tissues using Expression data) algorithm (16) is able to calculated immune scores to predict the infiltration of immune cells by analyzing specific gene expression signature of immune cells, which has become new indicator to distinguish “hot” and “cold” cancers for immune therapy (17). Therefore, we attempted to verify the difference of immune scores between the COAD and READ. The result showed that the immune scores of COAD were higher than READ (*P* = 0.0006, **Figure 3A**). To confirmed this phenomenon, we expanded the statistical samples including 172 COAD and 66 READ from TCGA and found a similar results (*P* = 0.0278, **Figure 3B**). We performed ssGSEA algorithm (18) to evaluate correlation between anti-tumor immunity and pro-tumor suppression in our samples, as showed in **Figure 3C**, the correlation in COAD (r = 0.91) exhibited more positive than READ (r = 0.72). Moreover, we observed the abundance of immune markers in both anti-tumor immunity and pro-tumor suppression were significantly increased in COAD, as compared to READ (**Figure 3D**), suggesting a negative feedback mechanism is strongly embedded in the immune regulation of COAD. Based on the immune scores, the cluster analysis of COAD and READ showed an up-regulation of multiple immune cells were found in COAD (**Figure 3E**), suggesting more intense immune activity existed in COAD than in READ.

### Identification of Complement C3 as the Core Protein of DEPs in COAD

**Figure 4:**
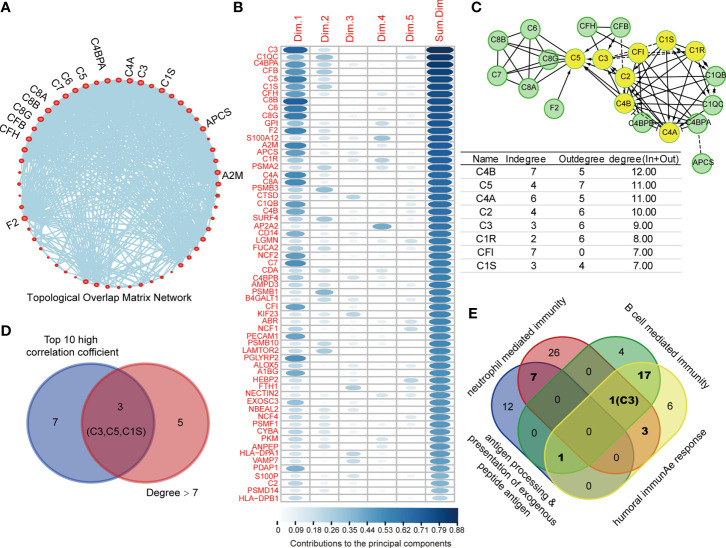
C3 is the core protein in topological overlap matrix (TOM) established by immune-relevant differential expressed proteins (DEPs). **(A)** 62 out of 77 immune-relevant DEPs were employed to establish the TOM, and those proteins with centrality higher than 30 were highlighted and named in the network. **(B)** 62 immune relevant DEPs were subjected to principal component analysis (PCA) analysis, the contribution values of individual protein to top five principal component were summarized and ranked. **(C)** The Protein-Protein Interaction network of the 62 immune-relevant DEPs were plotted by REACTOME (upper). The degrees for each protein in the network were listed (lower). C3, C5, and C1S showed not only degree > 7, but also ranked as the top 10 high correlation coefficient proteins **(D)**. **(E)** The Venn plot depicting the C3 was the only protein that co-identified in four immunity related pathways.

To identify the core protein of DEPs with high prognostic value for COAD, we performed topological overlap matrix (TOM) to investigate the hub protein using 77 DEPs in above-mentioned immune-relevant pathways. The dendrogram of 77 DEPs by dissimilarity coefficient calculated from TOM was built (**Figure S2A–C**). As shown in **Supplementary Figure S2D**, only 62 immunity-relevant DEPs were clustered and presented in the blue module, among which, 15 DEPs with centrality over 30 were highlighted and showed in **Figure 4A**. In addition, as the variables, the 62 immunity-relevant DEPs were subjected to PCA analysis (**Figure S3A, B, Table S4**). Consequently, the contribution values of 62 DEPs to principal components were displayed, showing C3 had the highest correlation (**Figure 4B**). In addition, the 62 immunity-relevant DEPs were subjected to REACTOME analysis, the protein-protein interaction network related to complement system was displayed in **Figure 4C**. The proteins with the degree (in and out) in the protein-protein interaction network over seven were highlighted (**Figure 4C**). Among the top 10 proteins with high correlation coefficient, we found that only C3, C5, C1S exhibited degree > 7 in the network (**Figure 4D**). Importantly, we found that C3 was the only co-identified protein in the progress of B cell mediated immunity, neutrophil mediated immunity, and humoral immunity response (**Figure 4E**). These data suggested that C3, one of the most crucial members in complement system with the function of activating complement pathway, is an effective indicator specifically for COAD.

### C3 Is Negatively Correlated With Overall Survival of COAD Patients

**Figure 5:**
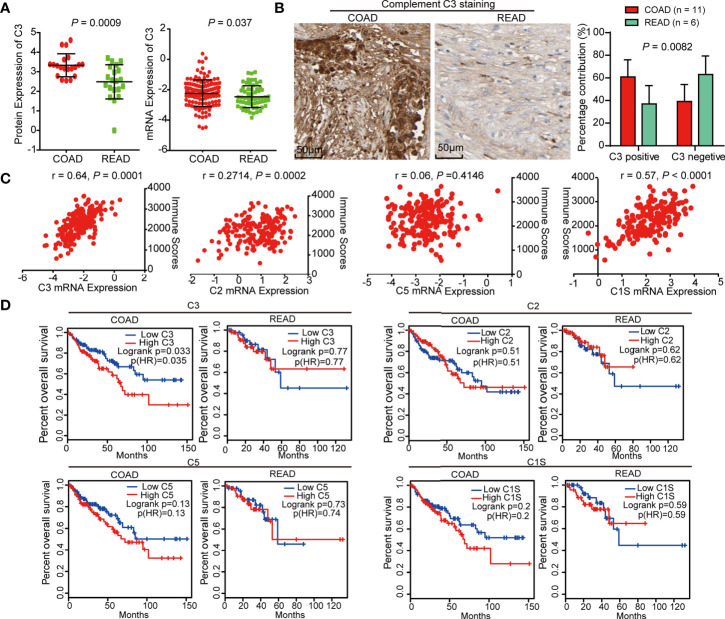
C3 is negatively correlated with overall survival of colon adenocarcinoma (COAD) patients. **(A)** The protein and mRNA level of C3 in COAD were higher than READ. **(B)** IHC staining of C3 in COAD and rectal adenocarcinoma (READ) using Human Protein Atlas, statistical analysis using IHC profiler plug-in of Image J software showed that C3 expression was significantly increased in COAD tissues as compared with READ. **(C)** The scatter plot showing the correlation between mRNA expression of C3, C2, C5, and C1S and immune scores, respectively. Only C3 exhibited significant correlation with immune scores. **(D)** Kaplan-Meier curves from GEPIA described that C3 expression is negatively correlated with overall survival of COAD patients but not READ. C2, C5, C1S expression were not significantly correlated with overall survival of both COAD and READ patients.

We further explored the clinical significance of C3 and found that the protein and mRNA level of C3 in COAD was higher than in READ (**Figure 5A**). IHC staining of C3 in COAD and READ tissue slices using Human Protein Atlas database showed that C3 expression was remarkably increased in COAD tissues (**Figure 5B**). Moreover, we determined the correlation between C3, C2, C5, C1S, and immune scores originated from 127 COAD and 63 READ, respectively. The result showed that C3 (r = 0.64) and C1S (r = 0.57) were positively correlated with immune scores, while C2 and C5 were not correlated with immune scores, suggesting C3 and C1S are effective indicators of immunity (**Figure 5C**). Finally, the survival curves from Gene Expression Profiling Interactive Analysis (GEPIA, http://gepia.cancer-pku.cn/) showed that high expression of C3 linked to poor overall survival of COAD patients (LogRank *P* = 0.035), but not in READ (LogRank *P* = 0.77). Though survival curves of C2, C5, and C1S had similar tendencies with C3 between the two cancers, their survival curves exhibited no significant difference in the two cancers (**Figure 5D**), suggesting C3 is a specific indicator with prognostic value for COAD.

## Discussion

COAD and READ are two diverse cancers with distinct clinical features, the current genetic and epigenetic investigations remained insufficiently to explain their malignant behaviors. As executioners of life, protein profiling can provide valuable insight to identify the prognostic biomarkers for distinguishing COAD and READ. In this study, DEPs relevant to immune activation were upregulated in COAD but not in READ. Among the cores of the immune-relevant DEPs matrix, C3 was upregulated in COAD that linked to poor overall survival of COAD patients but not in READ, highlighting that C3-mediated immune response is the main difference between COAD and READ.

Increasing studies reported that CRC is a heterogeneous disease. CRC shows various biological and clinical differences including embryonic origin, vascular supply and main physiologic function that significantly link to prognosis (19). In clinical, worse prognosis is frequently observed in right-side CRC, as compared with left-side tumors in patients with wild-type RAS (20). In addition, COAD and READ with distinct clinical responses and outcome were frequently reported in clinic, which raised a great interest to investigate their difference. In this study, we compared the DEPs between the two cancers and found that the DEPs were mainly enriched in immune response. Immune scores analysis supported that both pro-tumor suppression and anti-cancer immunity cells were all upregulated in COAD, indicating tumor microenvironment immune phenotype of COAD is stronger than READ. Accumulating evidences demonstrated that immune cells played an important role in the tumor microenvironment by controlling cancer progression, which were attractive therapeutic targets (21–23). Accordingly, we proposed that COAD and READ had different immune landscapes that lead to different prognoses and treatment responses. Recent studies reported that immune infiltration showed better performance than TNM stage under in some conditions (17). In addition, it was reported that immunity could influence chemotherapy and radiotherapy by increasing tumor-suppressor factor such as HMGB1 or enhancing the expression of PD-L1 to hamper anti-tumor immunity in different cancers (24). The association between immunity and cancer prognosis has been widely reported. A study reported that immune score was remarkably associated with GBM subtypes, of which mesenchymal subtype ranked the highest immune score compared with neural subtype and classical subtype. Moreover, high immune scores were also relevant to poor overall survival in GBM patients (25). Our comparative proteomics predicted that different immune activity between COAD and READ implied different prognosis, which provided a new method to judge prognosis and a new direction for preventing recurrence of cancers for clinic in colorectal cancer.

Human complement system is constructed by a cascade of serine proteases, its activation involves multiple steps that tightly regulated (26). As one of the most important parts of immunity, activation of complement system is recognized to contribute to cancers progression, which enhances tumor growth and increases metastasis (27). C3 is a crucial member of complement system. Recent study showed that C3 was upregulated in leptomeningeal metastatic models and necessary for cancer growth, as it could activate the C3a receptor in the choroid plexus epithelium to disrupt the blood-CSF barrier that allowed cancer cell growth (22), suggesting C3 is a predictive indicator for cancer with a strong clinical value. Though C3 was reported to associated with CD4+ and CD8+ T lymphocytes in lung cancer, however, a majority of studies suggested that C3 play an oncogenic role in multiple cancers (28). In this study, our unbiased data mining confirmed that C3 was not only highly related to immune activity in COAD, but also upregulated in COAD and associated with poor prognosis. However, this phenomenon was not found in READ. Though the significance of C3 was found in these clinical data, the detail mechanism need to be further investigation.

In conclusion, we performed data mining to compare the differences between COAD and READ in proteome and revealed immune response relevant proteins were specifically upregulated. among these proteins, C3 was upregulated in COAD and associated with poor overall survival of COAD patients but not in READ, suggesting C3-mediated immune response is key feature for distinguishing COAD and READ, which provided clue for clinical therapy.

## Data Availability Statement

Publicly available datasets were analyzed in this study. This data can be found here: http://proteomecentral.proteomexchange.org/cgi/GetDataset?ID=PXD015905.

## Author Contributions

YW, J-ZL, and JZ designed the experiments and analyzed results. J-ZL and YW carried out the experiments and wrote the manuscript. JZ, X-LL, L-YZ, and C-TW assisted with the analysis of mass spectrometric data. JZ and YW supervised the research and edited the manuscript. L-YZ collected the data and assisted with the analysis of mass spectrometric data. All authors discussed the results and commented on the manuscript. All authors contributed to the article and approved the submitted version.

## Funding

This work was supported by the National Natural Science Foundation of China (82002948), the Fundamental Research Funds for the Central Universities (11619303), the China Postdoctoral Science Foundation (2020T130252, 2018M643372), and the Guangdong Natural Science Research Grant (2019A1515010196, 2019A1515110597).

## Conflict of Interest

The authors declare that the research was conducted in the absence of any commercial or financial relationships that could be construed as a potential conflict of interest.
