# Supplementary material for: Comparative Proteome Identifies Complement Component 3-Mediated Immune Response as Key Difference of Colon Adenocarcinoma and Rectal Adenocarcinoma
Source: Front Oncol. 2021 Feb 15;10:617890. doi: 10.3389/fonc.2020.617890 (PMC7959172; doi:10.3389/fonc.2020.617890)
Supplement: Supplementary file 1 [file DataSheet_1.pdf]

Comparative Proteome identifies Complement component 3 - mediated immune response as key difference of colon adenocarcinoma and rectal adenocarcinoma

Jun-Ze Liang[1], Xiao-Ling Liang[1], Li-Ye Zhong[1], Chu-Tian Wu[2], Jing Zhang[1, 2]*, Yang Wang[1]*

[1] MOE Key Laboratory of Tumor Molecular Biology and Key Laboratory of Functional Protein Research of Guangdong Higher Education Institutes, Institute of Life and Health Engineering, College of Life Science and Technology, Jinan University, Guangzhou 510632, China;

2 Department of Gastroenterology, The First Affiliated Hospital, Jinan University, Guangzhou, 510630, China

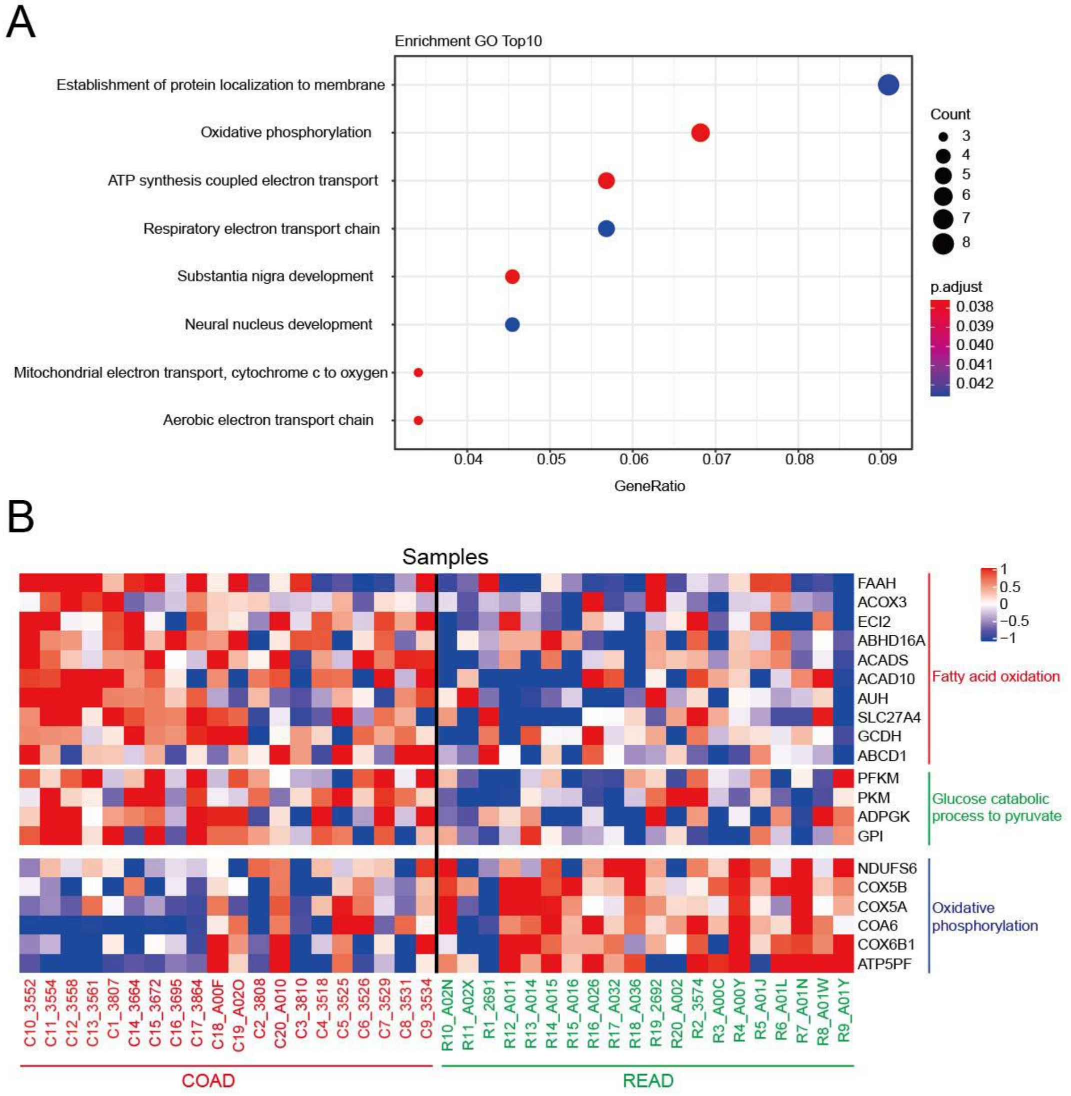

Supplementary Figure S1

Supplementary Figure S1. GO analysis of down-regulated DEPs in COAD. (A) GO analysis of the 92 down-regulated DEPs in COAD showing that these proteins were mainly enriched on "oxidative phosphorylation" and "ATP synthesis coupled electron transprot". **(B)** Heat map showing DEPs relevant to fatty acid oxidation and glucose catabolic process were globally upregulated, but oxidative phosphorylation associated proteins were decreased.

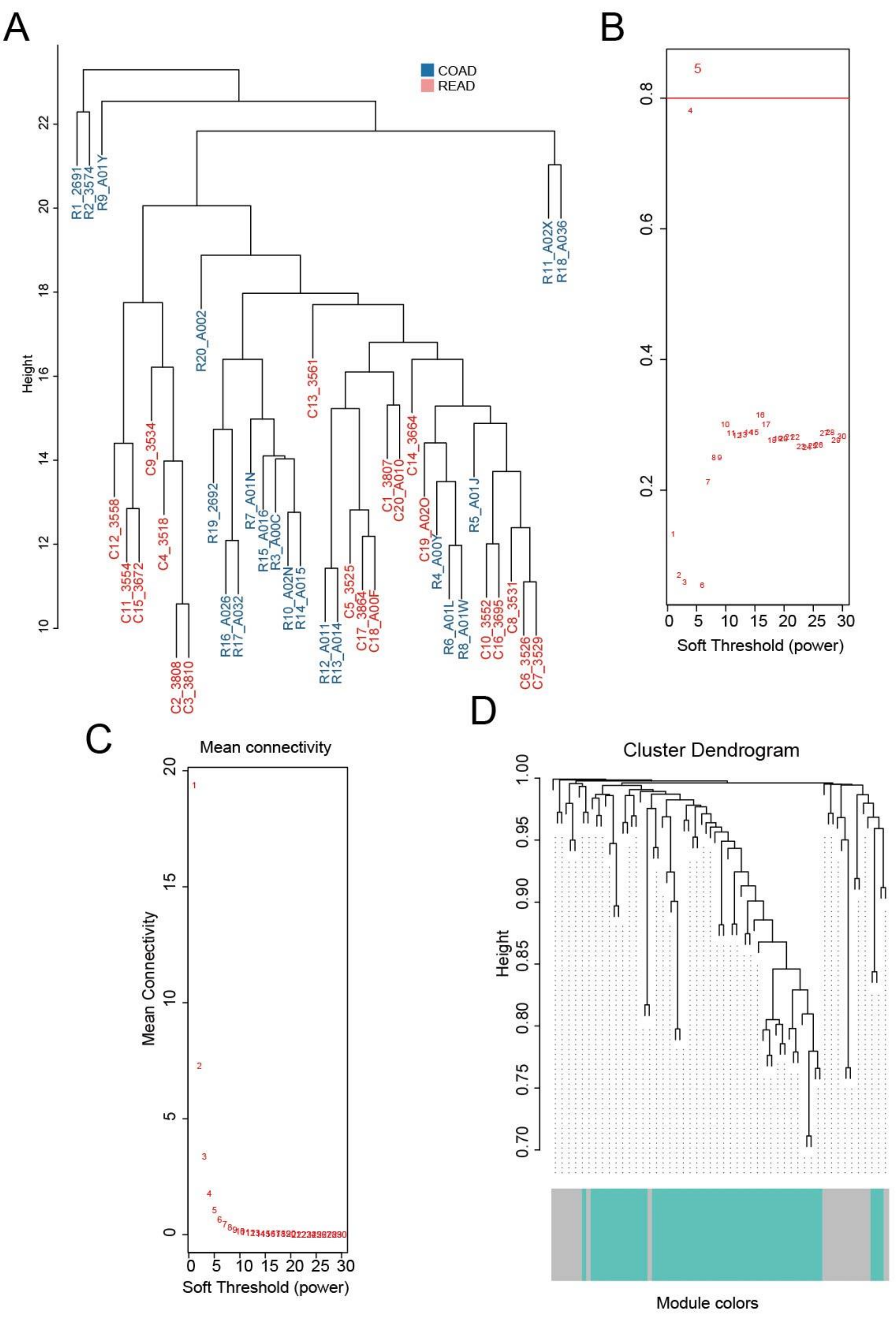

Supplementary Figure S2

Supplementary Figure S2. The establishment of TOM. (A) Hierachical cluster tree displaying several modules of DEPs. The samples of COAD and READ are designated by “Blue” and “Red”, respectively. **(B-D)** Weighted gene co-expression network analysis (WGCNA) of differentially expressed immune-related genes in the 40 CRC samples. Analysis of network topology for various soft thresholding powers, mean connectivity and non-scale network's goodness of fit in TOM with power of 1 to 30. High connectivity and model fit signified good TOM model. Power of 5 was Ultimately determined as the best power to build TOM (B-C). **(D)** Hierachical cluster tree displaying several modules of co-expressed genes. The two modules were validated and are designated by blue and gray.

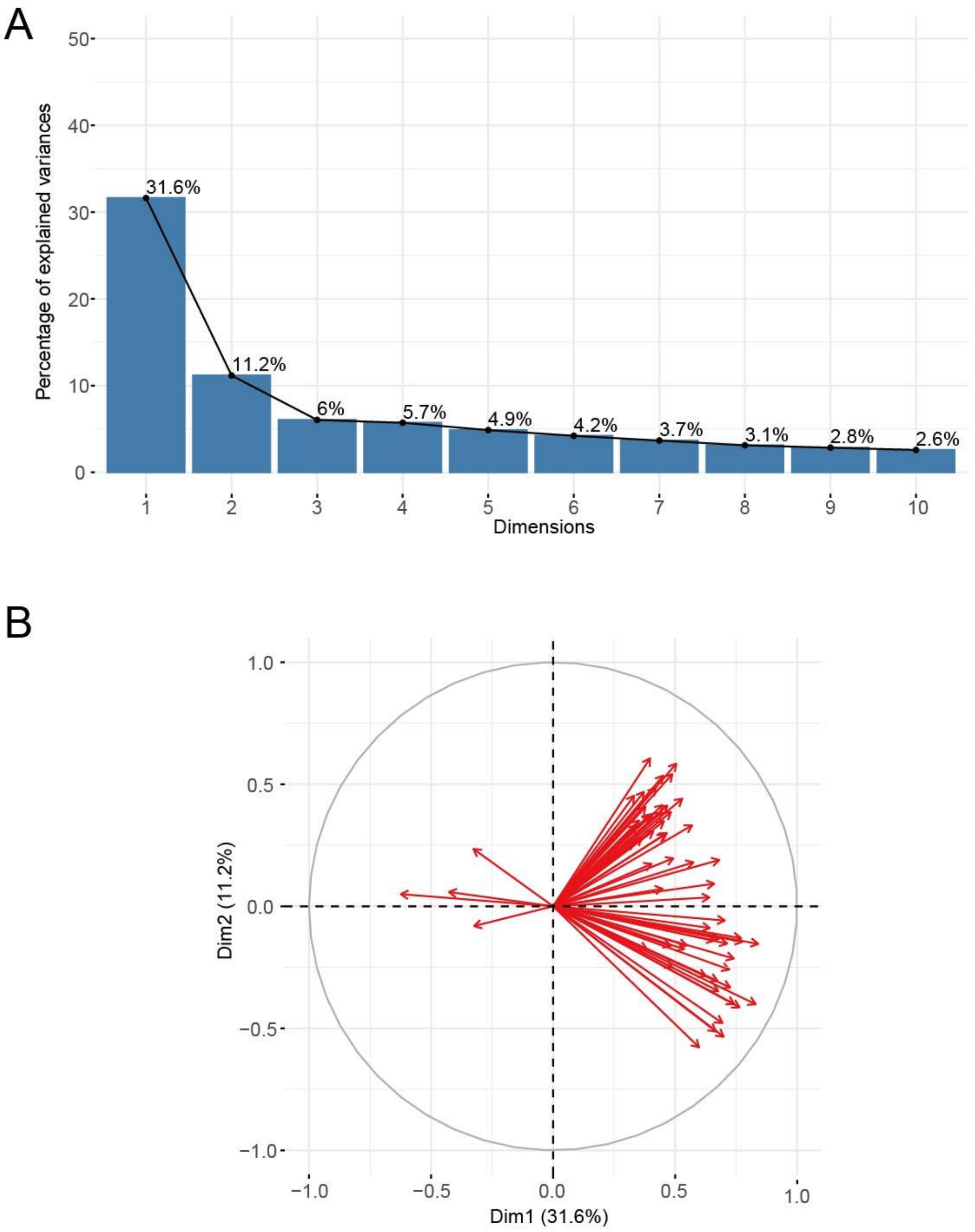

Supplementary Figure S3

Supplementary Figure S3. Principal component analysis of the proteomic data. (A) The 62 immunity-relevant DEPs were subjected to PCA analysis, the percentage of explained variances for each dimension were plotted (A). (B) The relation between dimension 1 (31.6%) and dimension 2 (11.2%) were plotted.
